# Elucidating the mechanisms of post-stroke motor recovery mediated by electroacupuncture using diffusion tensor tractography

**DOI:** 10.3389/fneur.2022.888165

**Published:** 2022-09-27

**Authors:** Min Su Kim, Byung Soon Moon, Jae-yoon Ahn, Sang-song Shim, Jong-Min Yun, Min Cheol Joo

**Affiliations:** ^1^Department of Physical Medicine and Rehabilitation, Soonchunhyang University Cheonan Hospital, Cheonan, South Korea; ^2^Professional Graduate School of Oriental Medicine, Wonkwang University, Iksan, South Korea; ^3^Department of Korean Internal Medicine, College of Korean Medicine, Wonkwang University, Iksan, South Korea; ^4^Department of Rehabilitation Medicine, Wonkwang University College of Medicine, Iksan, South Korea

**Keywords:** activities of daily living, acupuncture, cerebrovascular disorders, diffusion tensor imaging, gait, rehabilitation

## Abstract

Acupuncture has been commonly used for post-stroke patients, and electroacupuncture allows simultaneous application of acupuncture and electrical stimulation. We aimed to elucidate the mechanism of electroacupuncture on post-stroke motor recovery using diffusion tensor tractography. A total of 33 subacute stroke patients were recruited. The control group was subjected to conventional rehabilitation therapy. In contrast, the patients in the experimental group received electroacupuncture treatment for 30 min per session for 4 weeks in addition to the rehabilitation therapy. Fugl-Meyer assessment of the lower extremity (FMA_L), functional ambulation categories (FAC), and the Korean version of modified Barthel index (K-MBI) were used to compare behavioral outcomes between groups. The corticospinal tract (CST) was examined before and after the intervention *via* diffusion tensor tractography (DTT) to determine the motor recovery mechanism mediated by electroacupuncture. After 4 weeks of intervention, both the control and experimental groups showed a significant improvement with respect to FMA_L, FAC, and K-MBI. The level of improvement in FMA_L, FAC, and K-MBI did not vary significantly between the two groups. However, DTT results showed that the CST fractional anisotropy of the affected side (control: from 0.456 to 0.464, experimental: from 0.459 to 0.512) and its ratio (control: from 89.8 to 90.3, experimental: from 90.2 to 93.3) were significantly different between the two groups (*p* = 0.032 and *p* = 0.018). In addition, there were significant differences in the CST axial diffusivity of affected side (control: from 0.783 to 0.877, experimental: from 0.840 to 0.897) and its ratio variation (control: from 87.9 to 100.0, experimental: from 95.7 to 100.7) between the groups (*p* = 0.003 and *p* = 0.001). Electroacupuncture played a role in promoting brain plasticity and delaying neural degeneration in subacute period after stroke. Thus, electroacupuncture could be an effective adjuvant therapy in addition to conventional rehabilitation for motor recovery after stroke in a long-term perspective.

## Introduction

Stroke is the leading cause of mortality in South Korea and requires significant attention. The annual incidence of stroke is over 100,000 cases (1). Physical and psychological damage attributed to stroke is significant, and the disabilities attributed to stroke are observed across various locations of damaged lesions (2). To illustrate, well-known sequelae of stroke include hemiplegia, gait disturbance, language disorder, vascular cognitive impairment, and depression (3, 4). These aftereffects of stroke pose a significant challenge to the independence of the patient in daily routine and impose a significant burden on the family as well as the society (5, 6). To minimize such burden, rehabilitation therapy is performed at the hospital from the early days of the onset of stroke (7, 8), and a variety of evidence-based interventions are being used as adjuvants (9, 10). Among them, acupuncture has garnered significant interest.

Acupuncture involves the use of thin needles to stimulate specific pressure points linked to unwanted symptoms (11). Electroacupuncture, among various acupuncture techniques, is commonly used in combination with conventional stroke rehabilitation as it allows acupuncture and electrical stimulation to be simultaneously applied (12). The goal of electroacupuncture is to increase the potential therapeutic effects of standard acupuncture therapy (13). While acupuncture typically involves the use of a single needle on each point of interest during treatment, the modification in electroacupuncture involves the use of two needles (13). During the treatment, a weak current flows between the two needles that can mediate a stronger stimulation at the acupoint than the turning of the needle or other manual techniques used by the acupuncturist in general (11). For instance, the lower limb motor recovery in patients with stroke was reported to be more effective with the use of the combination of electroacupuncture and proprioceptive neuromuscular facilitation (PNF) rehabilitation therapy compared to that observed with the latter technique alone (14). In another study, the spasticity was significantly reduced when applied electroacupuncture (15). Nevertheless, there is a general lack of human studies investigating the mechanisms of motor recovery *via* electroacupuncture after stroke.

Diffusion tensor tractography (DTT), as a type of diffusion tensor imaging, is a technique that aids in the visualization of the 3D tracts of nerve bundles that are difficult to analyze with the conventional MRI (16). The DTT allows an in-depth analysis of the architecture and integrity of specific white matter tracts; hence, it is widely used in the prediction of post-stroke motor recovery and in the investigation of the mechanism of recovery (17). Therefore, the purpose of this study to investigate the therapeutic effects of electroacupuncture and to elucidate the mechanism of motor recovery mediated by electroacupuncture in subacute stroke patients using DTT.

## Materials and methods

### Subjects

The subjects in this study were subacute stroke patients diagnosed with ischemic or hemorrhagic stroke within 3 months. The diagnosis of stroke was performed based on BRAIN magnetic resonance imaging (MRI) or computed tomography (CT) performed at the emergency department by neurosurgeons or neurologists. The participants were between 18 and 80 years old and had moderate to severe motor weakness with a total score of Fugl-Meyer assessment 0–84. Patients with serious comorbidities or severe mental/cognitive disorders that are prohibited from using an electronic device in the body as part of electroacupuncture were excluded.

### Study design

This study was conducted as a double-blinded randomized controlled trial where the subjects were divided into two groups: patients in the control group received conventional rehabilitation therapy, and patients in the experimental group received electroacupuncture treatment in addition to the rehabilitation. The patients were randomized between the two groups using a random number table generated by a computer program, based on 1:1 allocation between the control and experimental groups. The random number table was prepared by an investigator who did not participate in the study registration.

### Electroacupuncture treatment

The patients in the experimental group received electroacupuncture therapy by acupuncturists. Standards for Reporting Interventions in Clinical Trials of Acupuncture (STRICTA) were followed for the intervention of electroacupuncture (18). The protocol for performing electroacupuncture therapy in this study was employed in previous studies (13, 19). Disposable sterile needles were used (stainless-steel needle, 0.30 × 30 mm, Woojin Medical Device Inc. Boryeong, South Korea) for acupuncture. The needle shaft had a thickness of 0.3 mm and a length of 30 mm. The insertion was performed up to the depth of 2–20 mm (20). After the insertion, the electroacupuncture (STN-330, Stratek, Anyang, South Korea) was connected to the needle handle on the affected side. Then, electrical stimulation was induced via a bipolar symmetric wave at a pulse width of 55 μs, frequency of 30 Hz, and interval mode (13). The electroacupuncture was performed for 30 min per session and five sessions a week, thereby completing 20 sessions during 4 weeks.

### Stroke rehabilitation therapy

Patients in the control group were subjected to conventional stroke rehabilitation therapy to restore motor function in the affected arm and leg and improve gait function. This conventional rehabilitation therapy was performed for 4 weeks with five sessions a week and 120 minutes per session. Besides, the control and experimental groups patients received stroke rehabilitation therapy equally, including physical and occupational therapy.

### Behavioral outcomes

To compare the treatment effects between the control and experimental groups, the lower limb Fugl-Meyer Assessment (FMA_L), the Functional Ambulation Categories (FAC), and the Korean version of the Modified Barthel Index (K-MBI) were used. The FMA_L is a tool widely used for the evaluation of the impairment of sensory-motor function in patients with stroke (21). It is a numerical scale with three scores per item, and the maximum score for lower limb motor function is 34 (21). The FAC categorizes gait patterns according to the level of assistance into six stages: a score of zero indicates nonfunctional ambulation, a score of 1 indicates ambulation dependent on physical assistance, a score of 2 indicates ambulation with intermittent help from one assistant, a score of 3 indicates ambulation without physical assistance but with guidance or monitoring, a score of 4 indicates independent ambulation on flat surface but dependent ambulation on steps or uneven surface, and a score of 5 indicates independent ambulation (22). In this study, the patients were divided into two groups, those with <3-point FAC and those with ≥3-point FAC based on the level of independent ambulation.

The K-MBI consists of ten evaluation categories (personal hygiene, taking a bath, eating a meal, relieving oneself, climbing steps, getting dressed, control of feces, control of urine, gait, and chair-to-bed movement) (23). Each category is evaluated on a 5-point scale with the application of nine weight values depending on the significance of the category content (23). The scores ranged from 0 to 100, where higher scores indicated more advanced ability to independently perform daily activities.

The data pertaining to demographic factors were also collected as they may influence motor recovery. The factors included age, gender, stroke risk factors (underlying diseases such as hypertension, diabetes, or hyperlipidemia), lesion location, lesion direction, and cognitive function (24).

### Diffusion tensor tractography analysis

The data pertaining to DTT were collected using the 3 Tesla MRI scanner. Using the single-shot diffusion-weighted echo-planar imaging sequence, a complete image of the brain was obtained from 33 patients enrolled in this study. The data set comprised 45 images with high diffusion weighting (b value = 1,000 s/mm^2^) applied along 44 diffusion directions and one image with no diffusion weighting (16). Each image included 60 2.25-mm thick axial slices of 1.96 × 1.96 mm in-plane resolution (16).

**Figure 1:**
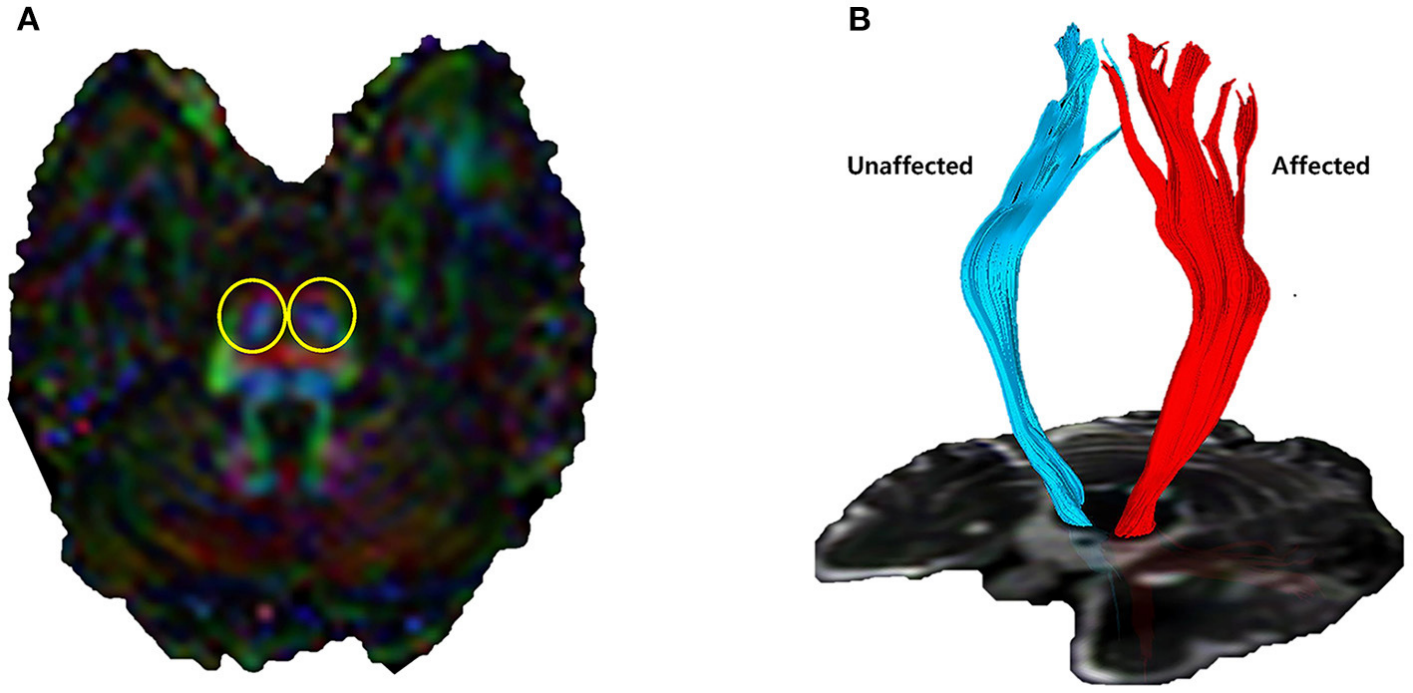
Corticospinal tract (CST) visualized by diffusion tensor tractography. **(A)** Lower anterior pons (yellow circles) and primary motor cortex were designated as regions of interest. **(B)** 3D reconstructed CST by fiber assignment by continuous tracking algorithm. Quantitative indicators such as the number of fiber tracts, fractional anisotropy (FA), and axial diffusivity (AD) can be identified.

Corticospinal tract (CST) fiber connectivity was evaluated using the fiber assignment by continuous tracking (FACT) algorithm and three-dimensional fiber reconstruction algorithm using PRIDE software (Philips Medical Systems, Best, the Netherlands). Termination criteria for fiber tracking were set as follows: fractional anisotropy (FA) < 0.2 and angle change >70° (25, 26). The two-region of interest (ROI) method was used for CST reconstruction, with ROIs including the motor cortex and lower anterior pons (Figure 1). We excluded fibers connected to the cerebellum (27). A quantitative analysis of ipsilateral CST parameters was performed by assessing FA, axial diffusivity (AD), and the number of fibers in the affected and unaffected CSTs. The FA ratio, AD ratio, and the ratio of the number of fibers were calculated by dividing the affected values by unaffected values, followed by multiplication of the resultant value with 100 (28).

### Statistics

To test the normality of the data pertaining to demographics and outcome measures, the Kolmogorov-Smirnov test was used. To analyze the differences in demographics between the control and experimental groups, the independent *t*-test was used for continuous variables and the chi-squared test was used for categorical variables. To examine post-intervention changes in result indicators, the paired *t*-test was used. To compare the treatment effects between the two groups, the independent *t*-test was used. To analyze the indicators of categorical variables, the chi-squared test was used. The level of significance was set as *p* < 0.05, and the SPSS ver. 22.0 software package (IBMSPSS, Armonk, NY, USA) for statistical analyses.

## Results

### Demographics and baseline characteristics

A total of 35 patients participated in this study. The data for two patients were excluded due to aspiration pneumonia in the middle of the treatment. Thus, the data of 33 patients (*n* = 17 for the control group and *n* = 16 for the experimental group) were analyzed. The mean age of the study participants was 63 yrs, and the mean treatment length for acute stroke determined between the onset and the first day of the intervention was 37.3 days (Table 1). No significant intergroup differences were observed with respect to age, gender, lesion direction, stroke type, lesion location, the interval between the onset and the first day of the intervention, and the state of cognitive function on the first day of the intervention. No side effect of electroacupuncture was reported throughout the intervention period.

**Table 1:** Baseline characteristics.

	**Control group** **(*n* = 17)**	**Experimental group** **(*n* = 16)**	***p*** **-value**
Age (yrs)	64.1 ± 9.1	62.0 ± 10.4	0.823
Gender (M: F)	10:7	11:5	0.469
Duration after onset (days)	36.4 ± 5.4	38.1 ± 6.2	0.672
Lesion side (Lt: Rt)	9:8	8:8	0.942
Location (supratentorial: infratentorial)	13:4	11:5	0.812
Comorbidity			0.782
Hypertension (%)	88	87	
Diabetes mellitus (%)	29	31	
Hyperlipidemia (%)	88	93	
MOCA	20.4 ± 5.4	19.2 ± 5.9	0.880

MOCA, Montreal Cognitive Assessment.

### Behavior outcomes

Before the intervention, no significant differences were observed in FMA_L, K-MBI, or FAC scores between the two groups. Following 4 weeks of intervention, FMA_L scores showed a significant improvement from 16.2 ± 7.3 to 22.0 ± 6.5 (*p* = 0.012) in the control group and from 16.7 ± 8.4 to 22.5 ± 8.6 (*p* = 0.014) in the experimental group (Table 2). The level of improvement between the two groups did not vary significantly (*p* = 0.630). The K-MBI scores also showed a significant improvement in both control and experimental groups after the intervention; K-MBI of the control group increased from 41.7 ± 17.1 to 66.7 ± 11.4 (*p* < 0.001) and that of the experimental group increased from 51.5 ± 15.1 to 71.2 ± 10.4 (*p* < 0.001). The level of improvement, however, did not vary significantly (*p* = 0.304).

**Table 2:** Comparison of behavioral outcome indicators in control and experimental groups after 4-weeks intervention.

	**Control group (*****n*** = **17)**		**Experimental group (*****n*** = **16)**		***p*** **-value^b^**
	**T0**	**T1^a^**	**T0**	**T1^a^**	
FMA_L	16.2 ± 7.3	22.0 ± 6.5^*^	16.7 ± 8.4	22.5 ± 8.6^*^	0.630
K-MBI	41.7 ± 17.1	66.7 ± 11.4^*^	51.5 ± 15.1	71.2 ± 10.4^*^	0.304
FAC					
<3	15	5	14	6	0.842^c^
≥3	2	12	2	11	

FMA_L, Fugl-Meyer Assessment of lower limb; FAC, Functional Ambulation Categories; K-MBI, Korean version of the modified Barthel Index (K-MBI).

* *p* < 0.05.

a Paired t-test for within group change.

b Independent t-test for between group comparison.

c Chi-square test for categorical data analysis.

The number of patients with <3-point FAC was 15 and the number of patients with ≥3-point FAC was 2 for the control group before the intervention. However, after 4 weeks of intervention, the number of patients with <3-point FAC was 5, and the number of patients with ≥3-point FAC was 12, indicating a remarkable change. For the experimental group, the number of patients with <3-point FAC was 14, and the number of patients with ≥3-point FAC was 2. A clear change was observed after electroacupuncture therapy; the number of patients with <3-point FAC was 6 and the number of patients with ≥3-point FAC was 11. The between-group comparison, however, did not show a significant difference in FAC scores depending on the treatment methods (*p* = 0.842).

### DTT outcomes

Through DTT, the CST fiber connectivity was analyzed before and after 4 weeks of intervention. With respect to baseline fiber numbers of the affected side, fiber ratio, FA of the affected side, FA ratio, AD of the affected side, and AD ratio, no significant differences were found between the control and experimental groups before intervention.

The CST fiber number of the affected side showed a significant decrease after intervention in both groups, with a decline from 586 ± 153 to 499 ± 139 (*p* = 0.012) in the control group and from 506 ± 157 to 431 ± 119 (*p* = 0.020) in the experimental group (Table 3). The fiber number ratio also showed a significant reduction after treatment in both groups, with a decrease from 58.1 ± 17.7 to 39.1 ± 10.6 (*p* = 0.008) in the control group and from 60.4 ± 27.8 to 39.2 ± 12.6 (*p* = 0.008) in the experimental group. The level of reduction, however, did not vary significantly between the two groups for both the CST fiber number and CST fiber ratio (p=0.442 and 0.256).

**Table 3:** Comparison of diffusion tensor tractography parameters in both groups after 4-weeks intervention.

	**Control group (*****n*** = **17)**			**Experimental group (*****n*** = **16)**			***p*** **-value^b^**
	**T0**	**T1**	***p*** **-value^a^**	**T0**	**T1^a^**	***p*** **-value^a^**	
Fiber No., affected	586 ± 153	499 ± 139	0.012^*^	506 ± 157	431 ± 119	0.020^*^	0.442
Fiber ratio^c^	58.1 ± 17.7	39.1 ± 10.6	0.008^*^	60.4 ± 27.8	39.2 ± 12.6	0.008^*^	0.256
FA, affected	0.456 ± 0.073	0.464 ± 0.081	0.082	0.459 ± 0.077	0.512 ± 0.088	0.004^*^	0.032^*^
FA ratio^c^	89.8 ± 14.5	90.3 ± 14.9	0.241	90.2 ± 14.1	93.3 ± 17.1	0.012^*^	0.018^*^
AD, affected	0.783 ± 0.105	0.877 ± 0.089	< 0.001^*^	0.840 ± 0.089	0.897 ± 0.112	0.032^*^	0.003^*^
AD ratio^c^	87.9 ± 11.7	100.0 ± 8.9	< 0.001^*^	95.7 ± 9.1	100.7 ± 9.8	0.036^*^	0.001^*^

FA, fractional anisotropy; AD, Axial diffusivity.

* *p* < 0.05.

a Paired t-test for within group change.

b Independent t-test for between group comparison.

c The ratio was defined as dividing the affected side by the unaffected side then multiplying it by 100.

The FA of the affected side (from 0.459 ± 0.077 to 0.512 ± 0.088, *p* = 0.004) and FA ratio (from 90.2 ± 14.1 to 93.3 ± 17.1, *p* = 0.012) in the experimental group showed significant improvement after four weeks of electroacupuncture therapy. There were no significant differences in FA and FA ratio of the affected side CST in the control group (*p* = 0.082 and *p* = 0.241). Besides, the patients in the experimental group demonstrated a more significant increase in the FA and the FA ratio than those in the control group after the intervention (*p* = 0.032 and *p* = 0.018).

The AD of the affected side showed a significant increase after intervention in both groups; an increase was observed from 0.783 ± 0.105 to 0.877 ± 0.089 (*p* < 0.001) in the control group and from 0.840 ± 0.089 to 0.897 ± 0.112 (*p* = 0.032) in the experimental group. For AD, the level of increase significantly varied between the two groups (*p* = 0.003). The AD ratio also showed a significant increase in both groups; an increase was observed from 87.9 ± 11.7 to 100.0 ± 8.9 (*p* < 0.001) in the control group after treatment and from 95.7 ± 9.1 to 100.7 ± 9.8 (*p* = 0.036) in the experimental group after treatment. The level of increment was significantly higher in the control group (*p* = 0.001).

## Discussion

The patients, either the control or experimental group, demonstrated significant improvement in behavioral outcomes, including motor function, gait capability, and activities of daily life after 4 weeks of intervention. Regarding DTT, the patients who received electroacupuncture therapy showed a more significant increase in the FA and the FA ratio and a lower increase in AD.

We investigated the effect of electroacupuncture on motor network plasticity with DTT, which was performed for the first time to our best knowledge. The DTT provides various parameters for the analysis of the white matter fibers. The degree of FA in a white or gray matter region reflects the structural integrity of white or gray matter in that region (29). The number of fibers calculated via DTT indicates the volume of voxels with FA above a threshold for each depicted tract (30). Because FA values within CST correlate positively with the degree of motor function improvement in patients with better recovery, the FA may serve as an imaging biomarker during the recovery process (29). In this study, CST-wise DTI analysis has shown significant interactions for the FA and FA ratio between intervention types and time by increasing the diffusivity across time for the electroacupuncture group. Our results showed that electroacupuncture therapy collaborating with conventional rehabilitation therapy could play a long-term positive role in restoring lower extremity motor function in subacute stroke patients.

In addition, the value of AD was significantly less increased in the experimental group than in the control group. AD refers to the magnitude of diffusion parallel to fiber tracts (31). A decrease in AD may indicate axonal damage in the acute phase after injury, whereas an AD elevation may be attributed to degenerative processes in the chronic phase (30). In this study, patients showed a gradual increase in AD for 4 weeks after the intervention of adjunct electroacupuncture treatment. The AD steeply decreased, however, in patients who received stroke rehabilitation therapy only. This finding may indicate that electroacupuncture through stroke rehabilitation therapy delayed the degenerative process during the transition to the chronic phase (30). This finding implies that long-term combined treatment would lead to better functional recovery in patients with stroke than stroke rehabilitation therapy alone.

We used the FACT algorithm to analyze the CST change in DTT, which was the core of elucidating the mechanisms of electroacupuncture effects in stroke patients. A whole-brain analysis is an exploratory approach that can be applied to investigate global white matter changes or whether such changes are heterogeneous across patients within a study (32). The most popular method is voxel-based analysis (VBA) which compares DTT metrics in every brain voxel (32). The FACT algorithm is an example of a VBA and can easily change discrete voxel information in a continuous track line (16). The line propagates in the vector direction of the pixels with discrete coordinates and can follow the actual tract more precisely (15). This strategy has high reproducibility, is time-efficient, and provides excellent spatially localized information based on the atlases coordinates (32). An alternative is running a cluster-based analysis and correcting them instead of correcting voxel-by-voxel. Tract-based spatial statistics (TBSS) overcomes issues about alignment and smoothing in voxel-based analysis by focusing registration and statistical testing exclusively on the center of the tracts (33). However, TBSS is known to suffer from several methodological limitations that complicate outcome interpretation (34). Because the FACT algorithm is easier to use for a DTT analysis than probabilistic algorithms like TBSS (35), previous studies have used a DTT analysis with the FACT algorithm to predict motor recovery (16, 36, 37).

Several theories have been proposed for elucidating the mechanism of nerve cell recovery mediated by electroacupuncture. First, electroacupuncture was reported to promote neurogenesis and cell proliferation in the central nervous system during the rehabilitation of patients with ischemic stroke (11). It can also control cerebral blood flow, inhibit apoptosis, and regulate neurochemical substances (13). In another study, electroacupuncture was shown to exert a neuroprotective effect and a neuroregenerative effect (38). These effects were reported to be based on mechanisms such as (1) regulation of oxidative stress, (2) suppression of glutamate excitotoxicity, (3) maintenance of the blood-brain barrier integrity, (4) inhibition of apoptosis, and (5) production of cell growth factors (39). Another study reported that electroacupuncture enhances the neuroplasticity in post-stroke subcortical regions and the cortical motor areas that compensate for the motor defect (40). These mechanisms are presumed to have led to the effects of electroacupuncture in preventing nerve cell degeneration, and based on this, the combined treatment is anticipated to induce functional recovery in patients with stroke in the long term.

There are several limitations to this study. As follow-up monitoring was not performed for the behavioral outcomes and DTT parameters after the completion of the intervention, the long-term effects of electroacupuncture should be addressed in the future study. The results have shown a significant difference in FA and AD between the control and experimental groups, and it is thus possible that among the parameters of behavioral outcomes, the lower limb motor function and gait function would have significantly improved in patients who received the electroacupuncture therapy had the study been conducted for a longer duration. In addition, this study was conducted with a small sample at a single institution for the assessment of behavioral outcomes and neuroimaging analysis. In the future, a study recruiting a higher number of subjects based on a longitudinal design should be conducted to clearly identify the effects of electroacupuncture on post-stroke lower limb motor recovery.

In summary, after 4 weeks of intervention, the lower limb motor function and gait capability significantly improved in patients subjected to conventional rehabilitation therapy alone and patients subjected to electroacupuncture. The DTT results provided evidence that electroacupuncture can promote brain neuroplasticity and delay the progression of neural degeneration over time. Thus, the treatment combining electroacupuncture with conventional stroke rehabilitation therapy could be a good protocol to promote motor recovery after stroke in the long term.

## Data availability statement

The original contributions presented in the study are included in the article/supplementary material, further inquiries can be directed to the corresponding author/s.

## Ethics statement

The studies involving human participants were reviewed and the study was conducted with the approval of the Institutional Review Board of the Wonkwang University Medical Center (IRB No. WKUIOMH-IRB-2020-14). The patients/participants provided their written informed consent to participate in this study.

## Author contributions

J-ML and MJ: conceptualization. J-yA, S-sS, and BM: methodology. J-ML and S-sS: formal analysis. J-yA, BM, and MK: investigation. MK: writing. J-ML: editing. MJ: funding acquisition. All authors have read and agreed to the published version of the manuscript.

## Funding

This study was funded by the Ministry of Health and Welfare and was supported by the Korea Health Industry Promotion Agency's Health and Medical Technology R&D Project (Grant Number: HI20C1951) and by the Soonchunhyang University Research Fund.

## Conflict of interest

The authors declare that the research was conducted in the absence of any commercial or financial relationships that could be construed as a potential conflict of interest.

## Publisher's note

All claims expressed in this article are solely those of the authors and do not necessarily represent those of their affiliated organizations, or those of the publisher, the editors and the reviewers. Any product that may be evaluated in this article, or claim that may be made by its manufacturer, is not guaranteed or endorsed by the publisher.
